# The long non-coding RNA PARROT is an upstream regulator of c-Myc and affects proliferation and translation

**DOI:** 10.18632/oncotarget.8985

**Published:** 2016-04-25

**Authors:** Dubravka Vučićević, Maja Gehre, Sonam Dhamija, Lennart Friis-Hansen, David Meierhofer, Sascha Sauer, Ulf Andersson Ørom

**Affiliations:** ^1^ Max Planck Institute for Molecular Genetics, Berlin, Germany; ^2^ Medizinische Hochschule Hannover Institute of Biochemistry, Hannover, Germany; ^3^ Department of Clinical Diochemistry, Næstved Hospital, Næstved, Denmark; ^4^ CU Systems Medicine, Würzburg, Germany; ^5^ Berlin Institute for Medical Systems Biology, Max Delbrück Center for Molecular Medicine, Berlin, Germany; ^6^ EMBL, Heidelberg, Germany; ^7^ RNA Biology and Cancer, German Cancer Research Center (DKFZ), Heidelberg, Germany

**Keywords:** MYC, long ncRNA

## Abstract

Long non-coding RNAs are important regulators of gene expression and signaling pathways. The expression of long ncRNAs is dysregulated in cancer and other diseases. The identification and characterization of long ncRNAs is often challenging due to their low expression level and localization to chromatin. Here, we identify a functional long ncRNA, PARROT (Proliferation Associated RNA and Regulator Of Translation) transcribed by RNA polymerase II and expressed at a relatively high level in a number of cell lines. The PARROT long ncRNA is associated with proliferation in both transformed and normal cell lines. We characterize the long ncRNA PARROT as an upstream regulator of c-Myc affecting cellular proliferation and translation using RNA sequencing and mass spectrometry following depletion of the long ncRNA. PARROT is repressed during senescence of human mammary epithelial cells and overexpressed in some cancers, suggesting an important association with proliferation through regulation of c-Myc. With this study, we add to the knowledge of cytoplasmic functional long ncRNAs and extent the long ncRNA-Myc regulatory network in transformed and normal cells.

## INTRODUCTION

The non-coding portion of the mammalian genome is pervasively transcribed resulting in the expression of thousands of mRNAs and long non-coding RNAs (ncRNA) [1, 2]. Although only a small number of long ncRNAs have been characterized to date they have been shown to be involved in almost every level of the gene expression program such as transcription, mRNA processing and translation [3]. Long ncRNAs are involved in the development of a variety of disorders such as cancer and neurodegenerative disorders [3, 4]. Long ncRNAs mediating regulation of tumor-suppressors and oncogenes through various mechanisms have been described [5-7]. The long ncRNAs lincRNA-p21 [8]; Pint [9]; PR-lncRNA-1 and PR-lncRNA-10 [5] are regulated by the tumor suppressor p53 and are involved in mediating its effects [6, 10]. Using a different mechanism, the long ncRNA-RB1 is co-expressed with RB1 from a bidirectional promoter and links RB1 transcription to the transcription of another tumor suppressor, Calreticulin, with effects on immunogenic cell death [7]. On the other hand, the long ncRNA ANRIL acts as an oncogene. ANRIL interacts with components of both the PRC1 and PRC2 complexes and mediates the silencing of the INK4a/ARF locus encoding tumor-suppressor genes that regulate cell cycle-progression and senescence [11]. Depletion of ANRIL leads to reduced proliferation and the long ncRNA is up-regulated in prostate cancer and leukemia further supporting an oncogenic role [12, 13].

The Myc family of proteins is a group of basic helix-loop-helixl eucine zipper transcription factors, one of the most studied classes of proteins associated to cancer. There are three members of the Myc family, c-Myc, n-Myc and l-Myc [14], all three functioning in a similar manner but with expression differences across cancers types. For instance, c-Myc is overexpressed in both blood-borne and solid tumors; n-Myc is expressed mostly in neural tumors; and l-Myc is overexpressed in small cell lung carcinomas [14-16]. Overall, many human cancers show increased expression of Myc. The transcription factor c-Myc regulates the transcription of at least 15% of the genes in the human genome with the help of its heterodimer partner MAX [17]. c-Myc is involved in many biological processes such as cell cycle, differentiation and protein synthesis [17, 18]. Since c-Myc is upregulated in many human cancers it is subject to intense investigation for cancer treatment [15, 16]. Several studies have shown that c-Myc regulates the expression of long ncRNAs, and that some of these transcripts can participate in the transcriptional functions mediated by c-Myc [19-22]. Long ncRNA-mediated regulation of translation of c-Myc has also been suggested [23, 24].

Here, we report that PARROT (proliferation associated RNA and regulator of translation) is a long ncRNA with a dynamic expression range across both transformed and normal cells that contributes to cellular proliferation in senescence and cancer and is associated to translation efficiency in HeLa cells. Using transcriptomic and proteomic approaches we identify PARROT as an upstream regulator of c-Myc. Depletion of PARROT leads to a depletion of c-Myc both at the mRNA and protein levels with subsequent effects on cellular growth and proliferation.

## RESULTS

### Differential promoter activity drives ncRNA expression

**Figure 1:**
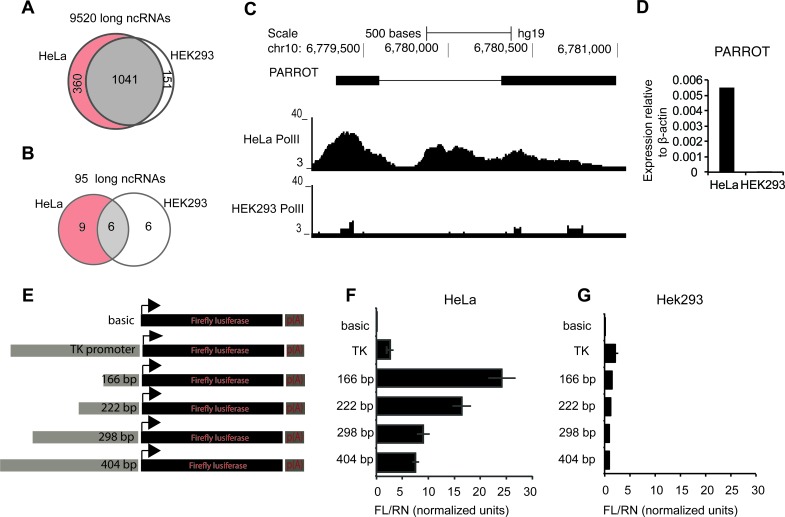
Differential promoter activity drives PARROT expression **A.** Pol(II) association to ncRNAs in HeLa and HEK293 cells. **B.** Pol(II) association to ncRNAs in HeLa and HEK293 cells differentially expressed upon keratinocyte differentiation. **C.** Snapshot from the genome browser of Pol(II) association to PARROT in HeLa and HEK293 cell line. **D.** Relative expression of PARROT in HeLa and HEK293 cells measured by qPCR. (**E**., **F**., **G**.) PARROT promoter region was cloned upstream of the Luciferase in pGL3 vector. **E.** Graphical representation of the inserts used. **F.**-**G.** Luciferase reporter assay. The Firefly luciferase vectors were cotransfected with a Renilla luciferase vector (pRL-TK) for transfection control in HeLa **F.** and HEK293 cells **G.**

Long ncRNAs are often expressed at a low level, complicating the further analysis of their transcriptional regulation and biological functions. Using available data for HeLa and HEK293 cells we identified the long ncRNAs with Pol(II) at their promoter in at least one of the two cell lines, to identify actively expressed and highly abundant long ncRNAs (Figure 1A). To reduce the number of candidates and to increase the probability of identifying long ncRNAs that are functional also in non-transformed cells, we restricted the analysis to long ncRNAs suggested to be functional based on their expression in keratinocytes and responsiveness to 12-O-Tetradecanoylphorbol-13-acetate (TPA)-induced differentiation of keratinocytes [25]. This results in a set of 21 long ncRNAs that show Pol(II) binding at their promoters in at least one of the cell lines (Figure 1B). While six long ncRNAs have defined Pol(II) peaks in both cell lines, most (15) are clearly bound specifically by high levels of Pol(II) in one cell line only as shown in Figure 1C, which is recapitulated in the expression level of the long ncRNAs in the respective cell lines, reflecting the tissue-specific properties of long ncRNAs that have mostly been reported for lowly expressed long ncRNAs (Figure 1D). While the generally low expression level of long ncRNAs is believed to result from post-transcriptional regulation, we asked whether the regulation of a highly expressed long ncRNA is indeed occurring at the promoter level by cloning an upstream region of the annotated transcript into a reporter vector (Figure 1E). We observed a high induction of PARROT expression in HeLa cells (Figure 1F), while essentially no transcription occurred in HEK293 cells (Figure 1G). Further characterization of the promoter by cloning of fragments of 166, 222, 298 and 404 base pairs, respectively, upstream of the transcription start site (TSS) in the same reporter assay identified a short promoter region mediating the tissue-specific expression of PARROT (Figure 1E-1G), similar to what is often observed for protein coding genes.

### PARROT is a spliced non-coding RNA localized to the cytoplasm

**Figure 2:**
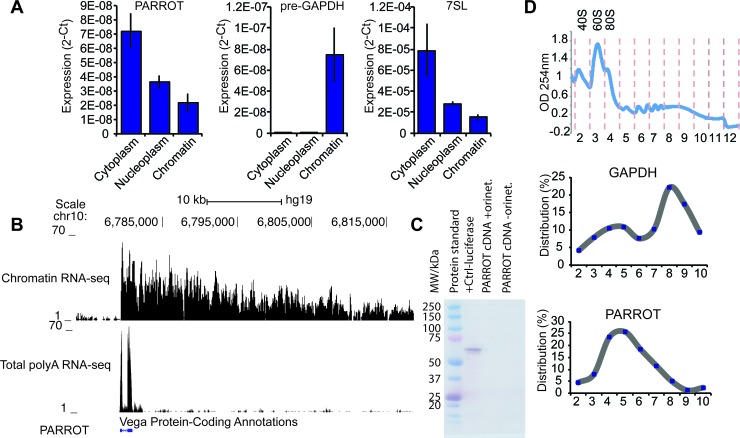
PARROT is primarily a cytoplasmic long non-coding RNA **A.** Distribution of PARROT in the cytoplasm, nucleoplasm and chromatin. As controls for the cellular fractionation pre-gapdh and 7SL are shown. The average of 2^−Ct^ ± s.d. are shown, *n* = 3. **B.** Snapshot from the genome browser showing the PARROT transcript in chromatin RNA-seq and polyA RNA-seq. **C.** *In vitro* translation assay. **D.** Polysome profile of HeLa cells. Polysomal distribution of GAPDH and PARROT RNAs was determined by isolating the RNA from each fraction collected from a 10-50% sucrose gradient. Percentage of mRNA level determined by qPCR in each fraction is shown.

To determine the cellular distribution of PARROT we subjected HeLa cells to cellular fractionation, separating the cells into cytoplasmic, nucleoplasmic and chromatin fractions [26]. We can show the localization of the processed form of PARROT to be primarily cytoplasmic (Figure 2A) suggesting functions different from regulation of transcription as has been reported for many nuclear localized long ncRNAs. We obtained the transcript profile of PARROT from chromatin-associated RNA using RNA sequencing (RNA-seq) with random primers and from whole cells using oligod(T) primers [27]. The transcriptome profile shows a very long primary transcript associated to chromatin and a polyadenylated, two-exonic processed transcript with a cytoplasmic localization as the spliced transcript form (Figure 2B), suggesting that PARROT might have dual functions as a long ncRNA in the nucleus as the primary transcript and in the cytoplasm as the processed transcript.

To confirm that the annotation of PARROT as a non-coding RNA is correct, we cloned the full-length processed transcript and synthesized RNA *in vitro* from a T7 promoter. *In vitro* translation assay analysis did not show the presence of any peptide translated from the long ncRNA sequence (Figure 2C). The localization of PARROT in polysome profiling experiments as determined by quantitative PCR (qPCR) of individual fractions is also suggestive of a non-coding function (Figure 2D). Furthermore, we confirmed that no peptides corresponding to the sequence of PARROT are detected in proteogenomic studies [28], as well as absence of association to ribosomes in ribosome profiling studies from HeLa [29].

### PARROT regulates proliferation and translation

**Figure 3:**
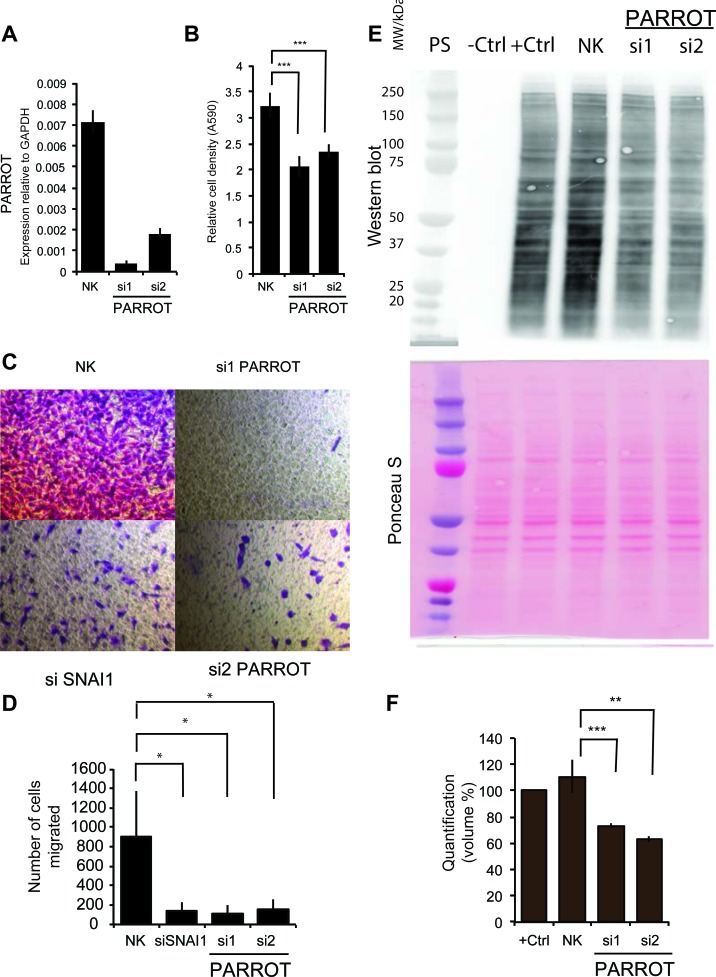
PARROT regulates growth and translation **A.** Knock-down of PARROT in HeLa cells with two different siRNAs. As a control, HeLa cells were transfected with a non-targeting control siRNA (NK). **B.** Crystal violet viability assay in HeLa cells transfected with either the control siRNA (NK) or depleted of PARROT for 72h. The average ± s.d. are shown, *n* = 5 (si1 *p* = 4.87581E-06, si2 *p* = 1.58272E-05). **C.** Migration assay. A549 cells were transfected with either the control siRNA (NK), two different siRNAs against PARROT or SNAI1which was used as a positive control, and **D.** quantified using three independent experiments. **E.** Puromycin translation assay with western blot using a puromycin antibody. Shown are: Protein standard (PS), protein extracts from control HeLa cells not treated with puromycin (-ctrl), control HeLa cells treated with puromycin (+ctrl), and cells treated with puromycin and transfected with either control siRNA (NK) or two different siRNAs against PARROT for 72h. F) Ponceau S staining as the loading control and the bottom panel: quantification of puromycin incorporation in three independent experiments. (The average ± s.d. is shown; ** *p* < 0.01, * *p* < 0.05).

To assess the function of PARROT we used siRNAs for knock-down in different cell lines. Following knock-down in HeLa cells (Figure 3A) a reduction in cell growth was observed using crystal violet staining (Figure 3B) that could not be assigned to apoptosis using a number of available assays (not shown). To address effects on migration, in addition to cell growth, we used A549 cells. A549 cells display reduced migration ability following knock-down of PARROT (Figure 3C). The effect on cellular migration is comparable to knock-down of SNAI1, a known regulator of cellular migration (quantified from three independent experiments in Figure 3D) [30], demonstrating a general function of PARROT in several cell lines and effects on both cell growth and migration. Due to the cytoplasmic localization of PARROT and its association to low-molecular weight polysome profile fractions, we propose that PARROT could have a regulatory function in translation or mRNA stability. To address this, we determined the effect of knock-down on general translation using a puromycin based translation assay. We see a markedly reduced translation observed in general upon knock-down of PARROT, as determined by puromycin incorporation and western blot of the newly synthesized protein (Figure 3E). The Ponceau S staining is used as a loading control showing total protein loaded in each lane, and three independent experiments were quantified to determine statistical significance (Figure 3F).

### PARROT is an upstream regulator of Myc

**Figure 4:**
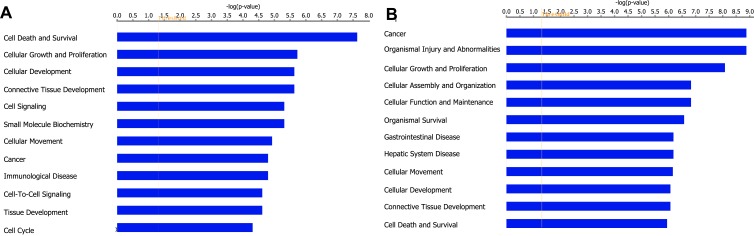
PARROT affects gene expression **A.** Gene ontology analysis of genes differentially expressed upon knock-down of PARROT. **B.** Gene ontology analysis of differentially phosphorylated proteins upon knock-down of PARROT.

To determine the molecular mechanism by which PARROT exerts its effect on the cellular phenotype we depleted it using siRNAs and performed RNA sequencing of polyadenylated transcripts in HeLa cells. This analysis reveals that the depletion of PARROT affects the expression of 331 genes of which 181 are upregulated and 150 are downregulated. Gene ontology analysis shows that PARROT affects the expression of genes involved in cell cycle regulation, cellular growth and proliferation as well as cellular movement (Figure 4A), in accordance with the phenotype observed following knock-down of PARROT in both HeLa and A549 cells.

To further explore the effect of PARROT on protein levels we used mass spectrometry to determine abundance and differential expression of the proteome following siRNA depletion of PARROT. Here, using SILAC (stable isotope labeling by amino acids in cell culture) control cells were labeled with heavy isotopes and the cells depleted of PARROT were labeled with light isotopes. Protein extracts were mixed in equal ratios and the intensities of both heavy and light labeled peptides were measured by mass-spectrometry. Analysis reveals that depletion of PARROT affects the levels of 73 proteins of which 22 are upregulated and 51 are downregulated. Furthermore, purifying phosphoproteins and subsequent identification of differentially phosphorylated proteins by mass spectrometry reveals that depletion of PARROT affects the phosphorylation status of 264 proteins: 98 are hyper-phosphorylated and 166 are hypo-phosphorylated. Gene ontology analysis shows that PARROT affects proteins involved in cell cycle regulation, cellular growth and proliferation as well as cellular movement on the level of protein synthesis as well as phosphorylation, in agreement with the results from RNA-seq data (Figure 4B).

**Figure 5:**
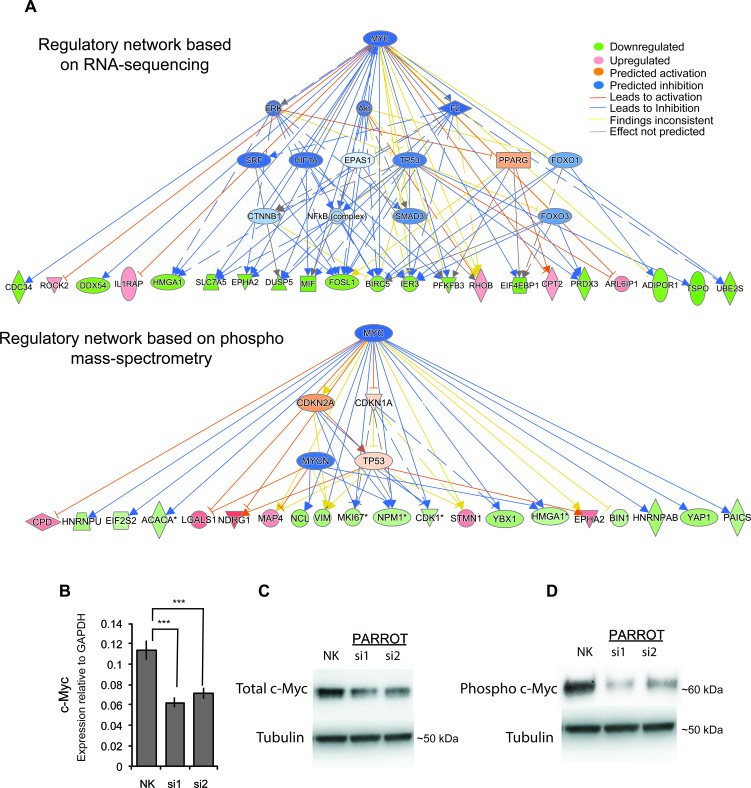
PARROT is an upstream regulator of c-Myc **A.** Ingenuity network analysis showing Myc as the prime target of PARROT. Regulatory network based on RNA-seq data (upper panel) or phospho mass-spectrometry data (lower panel) **B.** qPCR showing the levels of processed c-Myc transcript in HeLa cells transfected with either the control siRNA (NK) or depleted of PARROT. The average ± s.d. are shown, *n* = 3 (si1 *p* = 0.000541914, si2 *p* = 0.001295592). **C.**-**D.** Western blot of total c-Myc **C.** or phospho-form of c-Myc **D.** from protein extracts of Hela cells transfected with either the control siRNA (NK) or two different siRNAs against PARROT.

Incorporating all large-scale data into an Ingenuity Pathway Analysis (IPA) predicts c-Myc as a significant (*p* = 3.09×10^−5^) upstream regulator of the genes affected by the depletion of PARROT. Known Myc targets such as CDC34 (involved in cell death) [31], ROCK2 (involved in cellular adhesion, migration, apoptosis, differentiation) [32-34]; EPHA2 (migration, proliferation, adhesion, growth) [35]; RHOB (Ras family member involved in apoptosis, growth, proliferation, transformation, migration, adhesion) [36]; SLC7A5 (involved in growth and proliferation) [37]; DUSP5 (negatively regulates members of MAP kinase superfamily which are associated with cellular proliferation and differentiation) [38]; MIF (involved in proliferation, apoptosis, migration, aging and cell death) [39]; and FOSL1 (involved in proliferation, migration, motility, invasion, cell cycle progression, differentiation, apoptosis, etc.) [40, 41], are differentially expressed and/or phosphorylated following PARROT depletion (Figure 5A). We examined the direct effects of PARROT knock-down on c-Myc both at the RNA and protein levels. qPCR analysis reveals that the transcript level of c-Myc is decreased upon knock-down of PARROT (Figure 5B). In accordance with these results, the levels of both total c-Myc (Figure 5C) and phosphorylated c-Myc are decreased following knock-down of PARROT (Figure 5D). The regulation of c-Myc as an important target of PARROT is in agreement with the phenotypes in cell growth and migration observed since c-Myc is a known regulator of cellular proliferation and growth as well as of migration [17, 18].

### PARROT is associated with proliferation in senescence and cancer

**Figure 6:**
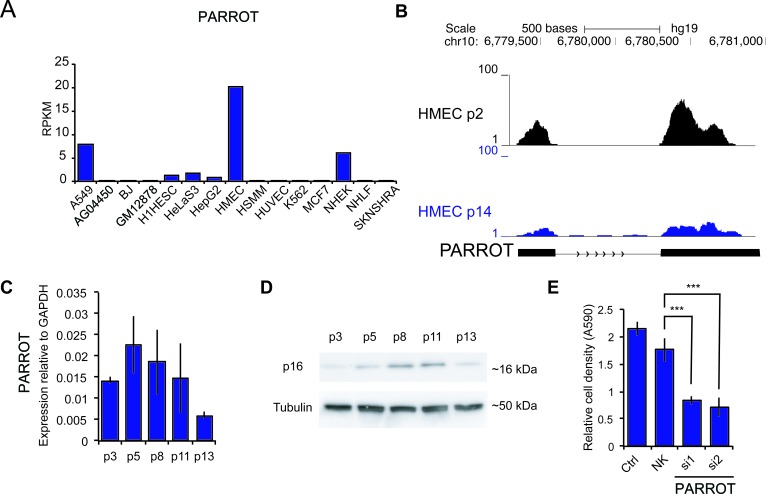
PARROT is differentially expressed in senescence **A.** RNA-seq data showing the expression of PARROT in different cell lines. **B.** Snapshot from the genome browser of RNA-seq data of HMECs cells in passage 2 and in passage 14. **C.** qPCR of PARROT in ageing HMECs. **D.** p16 western-blot in ageing HMECs. In C and D p refers to passage number. **E.** Crystal violet viability assay of HMECs cells in passage 5 of nontransfected cells (ctrl) or cells transfected with either control siRNA (NK) or two different siRNAs against PARROT for 72h. The average ± s.d. are shown, *n* = 5 (si1 *p* = 1.09588E-12, si2 *p* = 2.86182E-12).

The effect on translation and the association to proliferation together with the regulation of c-Myc prompted us to look at biological scenarios where proliferation is changed. An expression analysis of PARROT across a panel of cell lines shows differential expression of PARROT between cell lines. PARROT is expressed at the highest level in human mammary epithelial cells (HMEC) (Figure 6A). HMECs are healthy, untransformed, human mammary epithelial cells that undergo replicative senescence, an irreversible arrest of cell growth, after approximately 14 passages [42]. HMECs from young, proliferative (passage 2) and senescent (passage 14) cells were analyzed for PARROT expression by RNA-sequencing (Figure 6B) and qPCR (Figure 6C). As a marker of senescence we did western blot for p16 (Figure 6D). In accordance with the RNA-seq data, qPCR analysis shows that as HMECs grow older with passaging the expression of PARROT gradually decreases (Figure 6B and 6C). Knock-down of PARROT in HMECs decreases the cell growth significantly as we also observe in HeLa cells (Figure 6E), showing that PARROT is not only involved in proliferation of transformed cells, but has an effect on cell growth in non-transformed cells also.

**Figure 7:**
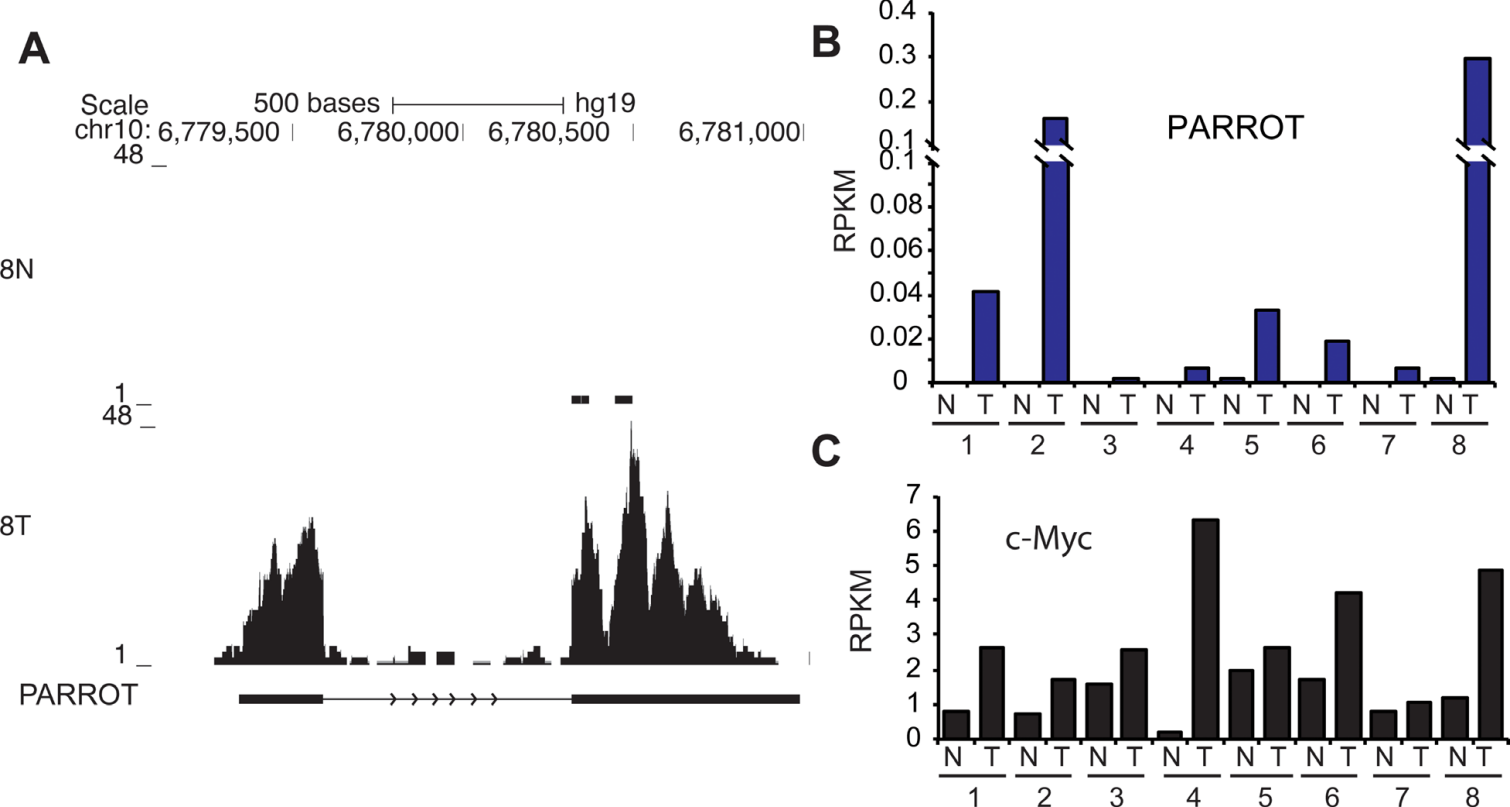
PARROT is upregulated in stomach cancer **A.** Genome browser snapshot of RNA-seq in control or tumor stomach cancer sample. **B.** Expression (RNA-seq) of PARROT in control (N) and tumor (T) stomach cancer samples. **C.** Expression (RNA-seq) of c-Myc in control (N) and tumor (T) stomach cancer samples. B and C show expression of PARROT and Myc, respectively, as RPKM (reads per kilobase per million reads) as quantified from RNA-seq data.

In the other end of the spectrum of proliferation, analysis of 8 paired normal and tumor samples from stomach shows that PARROT is not expressed in healthy stomach cells of stomach cancer patients. In tumor samples, on the other hand, it is highly expressed compared to normal tissue from the same patients (Figure 7A-7B). Furthermore, examination of the expression of c-Myc in these patients displays a similar trend, c-Myc is expressed at a significantly higher level in tumor samples than in normal paired tissue samples (Figure 7C). This observation supports our findings and further suggests that PARROT has an effect on proliferation that can lead to an oncogenic potential that is exerted *via* regulation of the c-Myc oncogene.

## DISCUSSION

The expression level of long ncRNAs is often very low and tissue-specific, properties that make the study of their functionality challenging. We have used data for Pol(II) association to long ncRNA promoters in two well-studied cell-lines, HeLa and HEK293 cells, to identify highly expressed long ncRNA with a pattern of differential expression between the cell-lines. We restricted the set of identified long ncRNAs by overlaying with data for differential expression of long ncRNAs in keratinocytes following differentiation, to reduce the number of candidates and increase the likelihood of studying functional long ncRNAs [25]. The low expression of most long ncRNAs is believed to be a consequence of rapid degradation. Using promoter assays, we can show that the high expression of PARROT in HeLa cells is a direct consequence of a strong promoter activity, and that the absence of PARROT expression in HEK293 cells is not caused by increased degradation but simply reflects that the promoter is not active in HEK293 cells. We identify a short core promoter reflecting properties comparable to most protein coding gene promoters, and the expression of PARROT in tumor samples suggests a specific transcriptional activation causing the overexpression of PARROT.

Interestingly, PARROT is transcribed as a very long primary transcripts and only a relatively short sequence in the 5′ end is further processed by splicing to the cytoplasmic two-exon mature form, suggesting that PARROT could have both nuclear and cytoplasmic functions. This might reflect a significant difference between processing of some long ncRNAs compared to protein-coding genes.

IPA upstream pathway analysis of PARROT targets identified independently by RNA sequencing or mass spectrometry, suggested c-Myc as one of the important upstream regulators, suggesting that PARROT is an upstream regulator of c-Myc. This hypothesis is supported by a robust effect of PARROT knock-down on c-Myc both at the mRNA, protein and phosphorylation level of c-Myc.

The pronounced effects we observe on proliferation, migration and translation in HeLa, HMEC and A549 cells following knock-down of PARROT are recapitulating the phenotype that would be expected for a decreased expression or activity of c-Myc [17, 43], and suggests a general function of PARROT in mediating upstream regulation of c-Myc in both transformed and non-transformed cells.

We can demonstrate an involvement of PARROT in senescence and cell proliferation in cancer that would be required for a regulator of c-Myc. We observe an increased expression of both c-Myc and PARROT in stomach cancer samples from eight patients analyzed by RNA sequencing. PARROT is dysregulated in 4 different types of cancer: BRCA (Breast Invasive Carcinoma), KIRC (Kidney Renal Clear Cell Carcinoma), LUAD (Lung Adenocarcinoma) and LUSC (Lung Squamous Cell Carcinoma) according to the lncRNome atlas (http://tcla.fcgportal.org). PARROT has previously been found to be associated with renal and lung adenocarcinoma [44] and could therefore be an important regulatory factor of the c-Myc pathway in several cancers as well as normal cells such as HMECs.

Several studies have shown that c-Myc can regulate the expression of long ncRNAs, and that some of these ncRNAs also participate in regulating the transcription of c-Myc target genes [19, 20, 22]. The promoters of long ncRNAs that are repressed by c-Myc have been shown to be more enriched for c-Myc binding sites than repressed mRNAs, suggesting a particularly important role for long ncRNAs in the Myc signaling network [21]. These long ncRNAs can act to regulate c-Myc targets and as oncogenes themselves promoting cellular proliferation and migration [19-22].

c-Myc directly binds to the promoter of H19 activating its expression by recruiting a histone acetyltransferase. A reduction in clonogenicity and anchorage independent growth is observed upon depletion of H19 in both breast and lung cancer cells [45]. These results suggest that H19 acts downstream of c-Myc to promote tumorigenesis in breast and lung cancer cells.

Similarly, in gastric cancer c-Myc has been shown to activate the expression of colon cancer associated transcript 1 (CCAT1). Overexpression of CCAT1 leads to an increase in proliferation and migration of gastric cancer cells [46]. A longer isoform of the same transcript called CCAT1-L has been shown to regulate the expression of Myc in colon cancer. It is proposed that CCAT1-L enables the interaction between the enhancer and the c-Myc promoter through modulation of CTCF concentration thereby promoting tumorigenesis [47].

A highly expressed long RNA in gastric cancer called gastric carcinoma high expressed transcript 1 (GHET1) also regulates c-Myc. This ncRNA enhances the stability and the expression of c-Myc by cooperating with insulin-like growth factor 2 mRNA binding protein 1 (IGF2BP1) leading to an enforced physical interaction between IGF2BP1 and c-Myc RNA. Depletion of c-Myc reduces the ability of GHET1 to promote proliferation of cancer cells [48].

We observe that the spliced form of PARROT is localized to the cytoplasm. There is a decrease in c-Myc mRNA as well as protein levels upon siRNA-mediated knock-down of PARROT, suggesting a role of PARROT in regulating mRNA stability or translation of c-Myc. The long ncRNA PCAT-1 has been suggested to regulate the translation of c-Myc through disruption of miRNA binding to the Myc 3′ UTR and the long ncRNA GAS5 has been shown to bind the translation initiation factor eIF4E and specifically target c-Myc translation [22, 24]. Our findings that PARROT can orchestrate a group of genes regulated by c-Myc identifies an additional factor in this complex network with important implications for both normal and cancer cells and the understanding of the molecular mechanisms involved.

Further investigation is needed to shed light onto the mechanism by which PARROT regulates c-Myc, which could reveal important mechanistic insight into how c-Myc is regulation modulated in both untransformed and transformed cells.

## MATERIALS AND METHODS

### Pol(II) association analysis

Long ncRNAs differentially expressed upon keratinocyte differentiation were obtained from [25]. Publicly available ENCODE Chip-seq data for Pol(II) were used. Long ncRNAs and Pol(II) were intersected by the use of BEDTools [49].

### Cell culture

HeLa, HEK293 and A549 cells were cultured in complete DMEM medium (Gibco/LifeTechnologies, Carlsbad, CA, USA) supplemented with 10% fetal bovine serum and penicillin-streptoMycin (Gibco). HMEC cells were cultured as previously described in a 1:1 mixture of MM4 and MCDB170 medium with of 5μg/ml of cholera toxin (Sigma), 5% AlbuMAX (Invitrogen) and 1μM oxytocin (Bachem) as described in [50]. All cells were cultured at 37°C with 5% CO_2_.

### Luciferase assay

Promoter upstream region (166bp, 222bp, 298bp and 404 bp) sequence located upstream of the PARROT TSS were cloned upstream of the luciferase into pGL3-basic vector (Promega). Primers used for amplification of the human genomic DNA with Phusion DNA polymerase (NEB) (Forward: 404bp- 5′-TGAAGATCTCTGACCACCTGTTGAGCTGT-3′, 298bp- 5′-TGAAGATCTAGGAAATTGGTCAAGGTTGC-3′, 223bp-5′-TGAAGATCTCCACTCAGTTATTTTTGTCTCTCA-3′, 166bp- 5′-TGAAGATCTAAGCTCCCAGAAATGTCAGC-3′; Reverse: 5′-TGAAAGCTTCCACCCAGAGTCAAGGAGAC-3′) contain added BglII (NEB) and HindIII (NEB) restriction sites. Both the vector and the insert were digested with BglII and HindIII restriction enzymes to allow directional cloning.

HeLa and HEK293 cells (10,000 cells/well) were plated in 96-well white plates 24 h before transfection. Cells were co-transfected with 0.1 μg pGL3-basic-promoter constructs and 0.02 μg of pRL-TK vector (Promega) using lipofectamine2000 (Invitrogen). Luciferase activity was measured 24 h after the transfection using the Dual-Luciferase Reporter Assay System kit (Promega) according to the manufacturer's instruction. For data analysis, Firefly luciferase activity was normalized to that of Renilla luciferase. All transfections were carried out in triplicates at least three times.

### *In vitro* translation assay

*In vitro* translation assay (Promega) was performed according to the manufacturer's instruction.

### Cellular fractionation

RNA was extracted from cellular fractionsprepared from ~4 × 10^6^ cells as described in [27].

### Crystal violet viability assay

HeLa or HMEC cells (10,000 cells/well) were plated in 96-well plates 24 h before transfection with siRNAs against PARROT or control siRNAs. Three days after the transfection cells where washed with PBS and fixed with methanol for 15min after which they were washed with water and stained with 0.1% crystal violet. After 20min cells were washed with water and dried. The following day 50 μl/well of 33% acetic acid was added and the absorbance at 590nm was measured. All transfections were carried out in triplicates at least three times.

### Quantitative real-time PCR

Total RNA was extracted from cells following the manufacturer's instructions with TRIzol (Life Technologies). Reverse transcription was carried out with High Capacity RNA-to-cDNA Kit (Life Technologies) and qPCR was performed using the Fast SYBR Green Master Mix (Life Technologies) on a 7900HT Fast Real-Time PCR System (Applied Biosystems, Foster City, CA, USA). The relative expression was calculated by normalizing to the Actin or GAPDH expression level as control housekeeping genes.

The following primers were used: PARROT (5′-CAGAACAGAGCCACCTCCAG-3′, 5′-GCACCGTCTGTTGTTCATTC-3′), c-Myc (5′-GCTGCTTAGACGCTGGATTT-3′, 5′-CCTCCTCGTCGCAGTAGAAA-3′), GAPDH (5′-GCTCTCTGCTCCTCCTGT TC-3′, 5′-ACGACCAAATCCGTTGACTC -3′),and β-actin (5′-CGACAGGATGCAGAAGGAG-3′, 5′-GTACTTGCGCTCAGGAGGAG-3′).

### RNA interference

dsiRNA oligos from IDTDNA (siRNA1 PARROT: sense 5′-GAAUGAAAGCACAGCACCAUCCUGGAA-3′, antisense 5′-CCAGGAUGGUGCUGUGCUUUCAUTC-3′; siRNA2 PARROT: sense 5′- GCUGAAUCAAGAUGCUGACUUCAGCAC-3′, antisense 5′-GCUGAAGUCAGCAUCUUGAUUCAGC-3′) were used. As a negative control we used DS NC1 (IDT DNA). Cells were transfected at a final dsiRNA concentration of 50 nM using Lipofectamine 2000 (Life Technologies) according to the manufacturer's protocol. Cells were collected 24 h or 48h after the transfection for RNA isolation and 72 h after transfection for western blot analysis.

### Sucrose gradient fractionation and RNA isolation

Cytoplasmic lysates of HeLa cells were prepared and subjected to centrifugation through linear sucrose gradients (10-50% sucrose) essentially as described previously [51]. siRNA transfected cells (3x 10cm plates per condition) were rinsed and scraped in ice-cold PBS containing 100μg/mL cycloheximide. All subsequent steps were performed in ice-cold conditions. Cells were pelleted and resuspended in extraction buffer (20 mM Tris-HCl, pH 8.0, 140 mM KCl, 0.5 mMDTT, 5 mM MgCl_2_, 0.5% Nonidet-P40, 0.1 mg/ml cycloheximide, and 0.5 mg/ml heparin), incubated 10 min on ice and clarified by centrifugation for 10 min at 12000g. Approximately 400μL of supernatant was layered onto a 12-ml linear sucrose gradient (10-50% sucrose (w/v) in detergent free extraction buffer) and centrifuged at 4°C in an SW40Ti rotor (Beckman, Palo Alto, CA) at 35,000 rpm without brake for 120 min. The gradients were collected into 12 fractions (1ml each), and absorbance profiles at 260 nm were recorded (ISCO, UA-6 detector). 0.1 volume of 3 M sodium acetate (pH 5.2) and 1 volume of isopropyl alcohol were added to the probes for overnight precipitation at −20°C. RNA was purified using total RNA isolation kit (Nucleospin RNA II, Macherey & Nagel) following the manufacturer's protocol. RNA concentration was determined, and the samples were stored at −80°C.

### Puromycin translation assay

Three days after the knockdown 1μM puromycin was added to the cells for 30 min. Cells were harvested in RIPA buffer, sonicated and boiled for 5min at 95°C in Laemmli buffer. 15μg of protein was loaded on a 4-12% Bis-Tris gradient gel (NuPAGE Novex) and transferred onto a PVDF membrane. Membrane was blocked in 5% milk and incubated overnight at 4°C with 1μg/ml anti-puromycin antibody (Kerafast). Membrane was washed, incubated with secondary HRP conjugated antibody and developed using ECL reagent (Pierce). To make sure that the equal amount of protein from puromycin treated cells was loaded on the gel the membrane was stained with Ponceau S protein dye prior to blocking.

### Migration assay

A549 cells were seeded in a 6 well plate (100000 cells/well) 4 h before transfection with siRNAs against PARROT, SnaiI or control siRNAs. 48 hours later cells were starved for 24h in a DMEM medium without FCS after which 50000 cells were added in DMEM medium without FCS to a migration chamber placed in a well of 24 well plate containing 750 μl of DMEM with FCS. 14 hours later cells where washed with PBS and cells that did not migrate were swiped. Cells that migrated were fixed with methanol for 15min after which they were washed with water and stained with 0.1% crystal violet. After 20min cells were washed with water, dried and number of migrating cells quantified.

### Western blot

Cells were harvested in RIPA buffer, sonicated and boiled for 5min at 95°C in Laemmli buffer. 15μg of protein was loaded on a 4-12% Bis-Tris gradient gel (NuPAGE Novex) and transferred onto a PVDF membrane. Membrane was blocked in 5% milk and incubated overnight at 4°C with total c-Myc (Santa Cruz Biotechnology sc-40), phosphor-c-Myc (Abcamab32029)or p16 (Santa Cruz Biotechnology sc-468) antibody. Membrane was washed, incubated with secondary HRP conjugated antibody and developed using ECL reagent (Pierce).

### RNA sequencing

Total RNA was extracted using TRIzol (Life Technologies). RNAseq libraries were prepared with the TruSeq RNA Sample Preparation Kit v2. Sequencing was performed on a HiSeq 2000 instrument (Illumina, San Diego, CA, USA) using paired-end sequencing (2 × 50 bp). Data was analyzed with the TRUP pipeline [52]. Differential expression analysis was preformed in edgeR Bioconductor package [53]. The cutoff for differentially expressed genes was set at FDR < 0.05. The RNA-seq data are deposited in GEO with the accession number GSE69842.

### Mass-spectrometry

HeLa cells used for MS were grown in SILAC medium. They were lysed and digested by trypsin under denaturing conditions as previously reported [55]. Peptides were purified with C18 columns and fractionated by a strong cation exchange (SCX, Polysulfoethyl A, 200 × 9.4 mm, 5μm 200Å, PolyC, Columbia, MA) column, 5% of resulting peptides in each of the 11 fractions were used for proteome profiling. The remaining peptides were further enriched for phospho peptides by TiO_2_ (GL Sciences, Japan) columns [55]. Each sample fraction was dissolved in 3 μL of 5% ACN and 2% FA and analyzed by LC-MS/MS. LC−MS/MS was carried out by nanoflow reverse phase liquid chromatography (Dionex Ultimate 3000, Thermo Scientific, Waltham, MA) coupled online to a Q-Exactive Plus Orbitrap mass spectrometer (Thermo Scientific) as described previously [54]. Raw MS data were processed with MaxQuant v1.4.1.2 [56] and searched against the human proteome database UniProtKB with 81.194 entries, released in 02/2012. A false discovery rate (FDR) of 0.01 for proteins and peptides and a minimum peptide length of 7 amino acids were required. A maximum of two missed cleavages was allowed for the tryptic digest. Following SILAC modifications were used: ^13^C_6_^15^N_4_-arginine and ^13^C_6_^15^N_2_-lysine. Cysteine carbamidomethylation was set as fixed modification, while N-terminal acetylation and methionine oxidation were set as variable modifications in all runs, phosphorylation of serine, threonine and tyrosine were set as variable modifications in the according enriched fractions.In order to filter for differentially regulated proteins or phosphoproteins we set the cutoff for the ratio of light (knock-down treatment) to heavy (control) to be > 1.2 for upregulated and < 0.8 for downregulated. Additionally, we also required that the ratio of knock-down/control to be > 1.2 or < 0.8 compared to the ratio of control knock-down(NK)/control.

### Gene ontology analysis

The functional analysis and networks were generated through the use of QIAGEN's Ingenuity Pathway Analysis (IPA, QIAGEN Redwood City, www.qiagen.com/ingenuity).

### Statistics

Statistical analysis was performed using two-tailed Student's *T*-test.
